# Faecal analyses and alimentary tracers reveal the foraging ecology of two sympatric bats

**DOI:** 10.1371/journal.pone.0227743

**Published:** 2020-01-16

**Authors:** Sydney Moyo, David S. Jacobs

**Affiliations:** Department of Biological Sciences, University of Cape Town, Rondebosch, South Africa; Senckenberg Gesellschaft fur Naturforschung, GERMANY

## Abstract

We used three complementary methods to assess the diet of two insectivorous bat species: one an obligate aerial hunter, *Miniopterus natalensis*, and the other *Myotis tricolor* whose morphology and taxonomic affiliation to other trawling bats suggests it may be a trawler (capturing insects from the water surface with its feet and tail). We used visual inspection, stable isotope values and fatty acid profiles of insect fragments in bat faeces sampled across five sites to determine the contribution of aquatic and terrestrial arthropods to the diets of the two species. The niche widths of *M*. *tricolor* were generally wider than those of *Miniopterus natalensis* but with much overlap, both taking aquatic and terrestrial insects, albeit in different proportions. The diet of *M*. *tricolor* had high proportions of fatty acids (20:5ω3 and 22:6ω3) that are only obtainable from aquatic insects. Furthermore, the diet of *M*. *tricolor* had higher proportions of water striders (Gerridae) and whirligig beetles (Gyrinidae), insects obtainable via trawling, than *Miniopterus natalensis*. These results suggest both species are flexible in their consumption of prey but that *M*. *tricolor* may use both aerial hawking and trawling, or at least gleaning, to take insects from water surfaces. The resultant spatial segregation may sufficiently differentiate the niches of the two species, allowing them to co-exist. Furthermore, our results emphasize that using a combination of methods to analyse diets of cryptic animals yields greater insights into animal foraging ecology than any of them on their own.

## Introduction

Knowledge of the diets of bats can provide baseline information on population ecology, foraging ecology, echolocation behaviour, home range size, nutritional needs and potential consequences of population declines [1]. Specifically, the analysis of bat diets may provide an indirect indicator of the foraging behaviour of bats, particularly because the nocturnal habits of bats make direct observation of their foraging behaviour in the wild difficult to obtain without expensive equipment.

As top nocturnal predators of insects, bats use a variety of foraging strategies [2,3] that are dependent on the kind of prey they hunt and the habitat in which bats forage. For example, species such as *Miniopterus natalensis* species are obligate aerial hawkers that catch prey in flight [4]. Additionally, many bat species may use more than one foraging strategy. For instance, some species (e.g. *Myotis capaccinii)* are known to switch between aerial hawking (catching prey in flight) and trawling (taking prey like arthropods and fish from or just below the surface of the water, respectively, using their hind feet; [5]). Several other *Myotis* species are also known to trawl for insects [6,7]. Generally, *Myotis* species typically hunt low over water, often seizing insects from the water’s surface using their feet [7]. However, the foraging behaviour of some *Myotis* (especially in the Southern Hemisphere) is still unknown particularly with respect to their ability to trawl for aquatic insects, with current reviews silent about the occurrence of trawling *Myotis* species in Africa [8].

Direct observation of the foraging ecology of bats would require the use of high-speed cameras [9,10]. Unfortunately, high speed cameras can be expensive [11] and often offer only instantaneous depictions of prey captured as opposed to providing information on prey assimilated by several individuals over several feeding bouts [12]. Visual inspection of faeces can also be useful in studying the foraging ecology of nocturnal, difficult to observe animals [13]. One method of indirectly assessing the foraging ecology of bats using faecal analysis, is to visually inspect the faecal material for fragments of insects. If the insects identified through such fragments can only be caught by bats using one kind of foraging method, then examination of faeces can also provide valuable insight on the foraging modes employed by bats. For example, pond skaters (Gerridae) and whirligig beetles (Gyrinidae) can only be caught by bats off the surface of the water and should fragments of these insects be found in the faeces of bats it would be an indication that those bats may potentially trawl or at least glean. Using bat faeces in this way has been a traditional and effective way of assessing the relative importance of insects to insectivorous bats [14].

Over the last two decades, faeces have also been used to investigate diets of free-ranging terrestrial species through stable isotope analysis [15–17]. Stable isotope analysis of faeces may offer greater accuracy and require less laboratory effort than visual faecal analysis [15]. Assimilation-based techniques such as stable isotope analysis have become promising tools in the examination of mammalian diets [18]. Using stable isotopes of carbon (Carbon-12 and Carbon-13) and comparing them to potential food sources, the original sources from which consumers obtained that carbon can be determined [19] because carbon-13 changes (fractionates) minimally (~ 0.5 ‰; [20]) from food to consumer. The use of stable isotopes in food web analysis has been used in several studies [13,17,21]. For example, stable isotopic values of carbon and nitrogen in faeces have been used successfully to demonstrate that eared moths (Noctuidae, Lasiocampidae and Geometridae) are very important to spotted bats (*Euderma maculatum*; [13]). The use of stable isotopic values of faeces to elucidate the foraging ecology of bats has also been advocated [16]. A possible way of using stable isotopes to study the foraging ecology of bats is to compare the dietary niche widths of species known to forage in the same area. This dietary niche approach has been successfully applied in other studies [21,22]. For instance, Whitaker [22] showed that species utilising similar foraging strategies will have similar dietary compositions and ultimately occupy similar dietary niches. Similarly, stable isotope ratios of hydrogen and nitrogen in two *Myotis species* known to trawl for insects in aquatic habitats differed from those of four *Myotis* species that gleaned insects in terrestrial habitats [21]. However, isotope values of different food sources can overlap introducing uncertainty into interpretations of stable isotope data [23]. For this reason, it is increasingly important for researchers to combine two or more methods to study the feeding ecology of animals [20,24]. While each approach has specific applications, strengths and weaknesses, a dual tracer approach is an innovative and promising way of overcoming the limitations of individual methods [25].

Fatty acid analysis represents an additional tool for the study of bat diets [17]. Fatty acids can be more specific to certain dietary sources than stable isotopes, removing some of the ambiguities that arise from using isotopes alone. Fatty acid analysis is premised on the knowledge that mammals do not synthesise physiologically important fatty acids in adequate amounts to meet their nutritional needs and must obtain it from their food sources [26]. The fatty acid needs of animals have been extensively studied in aquatic systems [26], but less research has been conducted to assess the fatty acid profiles of bat diets in both aquatic and terrestrial habitats (but see Lam et al. [17]). Like most aquatic animals, mammals, like bats, are unable to synthesise polyunsaturated fatty acids in adequate quantities to meet their physiological needs and must therefore derive these from their food. The most important polyunsaturated fatty acids (PUFAs), commonly termed physiologically important fatty acids [e.g. Eicosapentaenoic acid (EPA; 20:5ω3), docosahexaenoic acid (DHA; 22:6ω3)] are mainly produced in aquatic systems [27]. Because of the differences in physiologically important fatty acids between terrestrial and aquatic animals, fatty acids are useful in tracing the contributions of terrestrial versus aquatic prey to bat diets. However, despite the potential of fatty acids to elucidate the relative contributions of aquatic (characterised by 20:5ω3 and 22:6ω3) and terrestrial (characterised by 18:2ω6 and 18:3ω3) insects to the diets of bats few studies have used fatty acids in faeces; to our knowledge only one study [17] has done so and only for European *Myotis* species.

Bats can be assigned to guilds based on e.g. their foraging mode and habitat [28]. Bats from different guilds differ in both their habitat and foraging modes and do not therefore compete. Sympatric bat species that belong to the same guild (e.g. edge space aerial foragers), however, have to differ in at least one niche dimension to minimize competition among them. Ways in which niches could differ is through spatial segregation and/or the use of more than one foraging mode[28–31]. Sympatric bats belonging to the same broad guild may be able minimize competition within the guild by differentiating their niches through the use of more than one foraging mode allowing them to use space not utilized by other members of the guild foraging in the same habitat.

Here we used three methods of faecal dietary analysis to infer the diets of two insectivorous African species, *M*. *tricolor* and *Miniopterus natalensis*. These two species use the same habitats and roosts and may differentiate their niches through using slightly different foraging modes. *Miniopterus natalensis* has been characterised as an obligate aerial insectivore [4] and *M*. *tricolor* as using two foraging modes aerial hawking and gleaning [7]. Anecdotal observations, e.g. wet feet and tail, suggests that *M*. *tricolor* may glean or trawl aquatic insects from water surfaces (DS Jacobs, personal observations), sufficiently differentiating the niches of the two species to permit them to co-exist. We used visual inspection of prey remains in faeces as well as stable isotope and fatty acid analyses of faeces to: (1) quantify the relative contributions of aquatic and terrestrial insects to the diets of the two species, (2) compare the niche width of *M*. *tricolor*, which may both aerial hawk terrestrial insects and trawl/glean aquatic insects, with that of the obligate aerial hawker, *Miniopterus natalensis* [32,33]. We expected that the use of two foraging modes may give *M*. *tricolor* access to a wider range of insect prey resulting in it having a wider niche width than *Miniopterus natalensis*.

## Methods

### Model species

*Myotis tricolor* is a medium-sized insectivorous bat (~ 16g; [34]) that occurs across the eastern part of Africa, from Ethiopia to the southern part of South Africa [35]. *M*. *tricolor* has traditionally been categorized as an aerial hawking or a gleaning species [7], however, Stoffberg and Jacobs [34] could not induce *M*. *tricolor* to glean in captivity under a variety of circumstances.

*Miniopterus natalensis* is a medium sized species weighing about 12.5 g and distributed widely over southern Africa [36]. This species uses broadband echolocation dominated by frequency modulated calls [34]. *Miniopterus natalensis* is known to be an aerial hawker that forages in both open and cluttered habitats [4].

### Ethics statement

The capture and handling of all animals in this study and all methods of data collection complied with the guidelines recommended by the American Society of Mammalogists [37,38]. All workers handling bats were vaccinated for rabies and were required to use protective gloves when handling bats and samples. This project received ethics clearance from the University of Cape Town Animal Ethics Committee (UCT ethics clearance 2017/V11/Moyo). All bats and insects were sampled on both privately and publicly owned land after obtaining prior permission from owners or managers and the necessary permits from the relevant provincial nature conservation departments of South Africa (permit numbers: MPB 5590, 0056-AAA007-00216, OP 3654/2017 and OP3646/2017).

### Study sites

**Fig 1:**
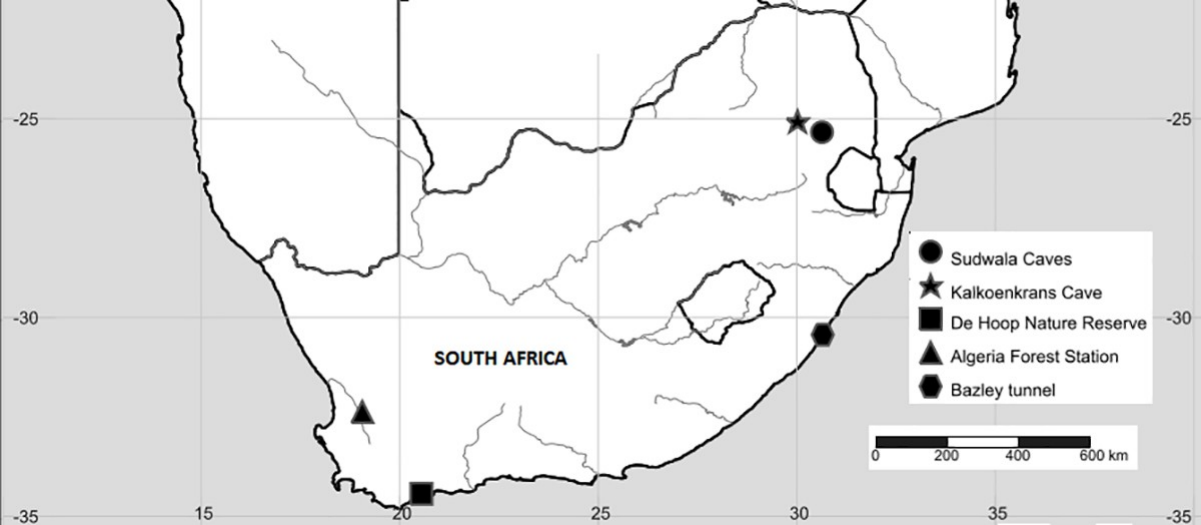
Map of faecal sampling stations (5 sites) for *Myotis tricolor* and *Miniopterus natalensis* between March and October 2017. Arthropods were also collected from the five sampling locations. The map was created using GPS coordinates in SimpleMappr (http://www.simplemappr.net/) and edited in CorelDRAW X7.

We selected five study sites where both *M*. *tricolor* and *Miniopterus natalensis* are reported to occur (summarised in Table 1; Fig 1). Sample collections occurred during the austral summer in De Hoop Nature Reserve (Overberg Region; February 2017), Algeria Forestry Station (Cederberg Region; April 2017) in the Western Cape Province, South Africa. We also collected samples in the austral spring (October 2017) in Mpumalanga Province (Kalkoenkrans and Sudwala) and Kwazulu Natal Province (Bazley tunnel).

**Table 1:** Descriptions of sampled sites for *Myotis tricolor* and *Miniopterus natalensis*.

Site name	Location	Ecological property	Dominant vegetation
De Hoop	34° 27′S, 20°26′E	Proximity to vlei (ephemeral lake) filled with water	coastal fynbos dominated by restios [65]
Algeria Forest	32° 22′S, 19°03′E	Proximity to large water source	Fynbos vegetation[41]
Kalkoenkrans	25°4′S, 30°1′E	Proximity to small river	Woodland[90]
Sudwala	25°22′S, 30°42′E	Occurs close to a narrow stream with many riffles	Arid grassland[41]
Bazley	30°26′S, 30°39′E	Far from large water source	Coastal forest with low shrubs [90]

### Collection of faeces

Bats were captured (one sampling occasion at each site) as they returned to roosting sites after foraging. We used a combination of mist nests and harp traps to capture bats (10 bats per site; n = 5 males and n = 5 females, where possible). All bats captured during sampling occasions were weighed (using a portable electronic scale), sexed, and aged. Aging of bats ensured that only adult bats were collected. Pregnancy status of females were identified by palpation of the abdomen using a palpation guide [39]; no pregnant females were analysed. Juveniles were identified by the presence of cartilaginous epiphyseal plates in the finger bones [40]. We assessed the presence/absence of cartilaginous epiphyseal plates by trans-illuminating the extended wings of the bats with a head lamp. Only adult bats were used in subsequent analyses. Each bat was given a unique identifier (sample number) and placed individually into a clean soft cloth bag for a few hours. Leaving the bats in cloth bag for a few hours enabled the collection of fresh faecal material. Faecal material obtained from the cloth bags were labelled and stored at -80°C in aluminium foil envelopes or Eppendorf tubes until further analysis.

### Collection of potential food sources for bats

Previous studies indicate that *M*. *tricolor* feeds predominantly on Coleoptera but also consumes some Hemiptera, Hymenoptera, Neuroptera and Diptera [34,41]. Similarly, *Miniopterus natalensis* consumes Diptera, Hemiptera, Coleoptera, Lepidoptera and Isoptera [41,42]. To this end, we aimed to collect these insects and other dominant arthropods from each study site.

Because all insect capture methods are biased toward catching prey of a certain size, mass, or flight behaviour [43], a combination of bucket style light traps (Universal Black Light trap, BioQuip Products, Inc., Rancho Dominguez, California) and sweep nets (with 0.5 m diameter and a 1 m handle) were used for collecting potential prey items. Light traps are also effective at attracting many terrestrial insects (e.g. beetles and moths) and aquatic insects (e.g. mayflies and caddisflies) [43]. Light traps were deployed simultaneously with bat faecal collections, however, we ensured that light traps were deployed far enough from bat sampling sites so that bat foraging behaviour was not influenced by the light traps.

Potential food sources of insect prey were also sampled to obtain reference isotope and fatty acid levels from the original source. These food sources included benthic algae and leaves from terrestrial plants [44]. Benthic algae (i.e. epiphyton, epipelon, epilithon, filamentous algae) were collected using a toothbrush and filtered onto pre -combusted (450°C for 5 hours) Whatman GF/F glass fibre filters (pore size = 0.7 μm). Terrestrial leaves were collected using a scalpel and scissors. All leaves were washed with distilled water before further processing.

### Laboratory protocols for extracting isotopes and fatty acids

All samples (in aluminium foil and Eppendorfs) intended for stable isotope and fatty acid analyses were freeze dried at -60°C for 48hrs to 72hrs (Virtis Consol 4.5 freeze dryer, SP Scientific Inc., Gardiner, NY, USA and New Brunswick Scientific Co., Edison, NJ, USA). After lyophilization, all faeces, insects, and plants (80–100 mg) destined for fatty acid and stable isotope analysis were ground to a fine, homogeneous powder and weighed using precleaned mortars and pestles.

Faeces (1–2 pellets; 0.5–0.7 mg) and insects (0.45 to 0.55 mg) were placed into tin capsules for stable isotope analysis. All samples were prepared, and the isotope ratios were analysed at the Archaeometry Laboratory in the Department of Archaeology, University of Cape Town. The δ^13^C and δ^15^N values of the plant and animal samples were determined on the IRMS [Elemental analyser (Carlo Erba) coupled to a continuous-flow isotope ratio mass spectrometer (Finnigan-MAT 252)] by comparisons with in-house laboratory standards [i.e. ammonium chloride (δ^13^C = -18.85‰, δ^15^N = 4.31‰), valine (δ^13^C = -27.92‰, δ^15^N = 12.10‰), acacia (δ^13^C = -29.34 ‰, δ^15^N = -1.11‰), sucrose (δ^13^C = -11.61‰), Merck gel (δ^13^C = -21.49‰, δ^15^N = 7.18‰), chocolate (δ^13^C = -18.72‰, δ^15^N = 4.17‰)] calibrated against International Atomic Energy Agency (IAEA) standards. Sample precision based on repeated sample and reference material was 0.1‰ and 0.15‰, for δ^13^C and δ^15^N, respectively. All values are reported in the δ^13^C and δ^15^N notation as parts per mille (‰) relative to international standards (PeeDee Belemnite for carbon and atmospheric N_2_ for nitrogen). The ratio of stable isotopes was expressed by convention in delta (δ) notation:

$$\delta =\left(\frac{R_{sample}}{R_{standard}}-1\right)*1000$$

where δ denotes the isotope ratio of the sample relative to the standard, and R_sample_ and R_standard_ are the ratios of heavy to light isotopes in the sample and the standard, respectively. One is deducted from the R_sample_/R_standard_ fraction so that samples with lower ratios of heavy isotopes than the standard are assigned a negative value and those with higher ratios of heavy isotopes than the standard have positive values. The resultant value was then multiplied by 1000 so that the ratio values were expressed in units of parts per thousand (‰).

Fatty acids were extracted from freeze-dried faeces (2–4 pellets per bat; 10–20 mg) and food sources (leaves and algae) by use of a modified one-step method [45]. Briefly, fatty acids were obtained using a process that included extraction of fatty acids with chloroform (containing 0.01% butylated hydroxytoluene) in a mixture of methanol and sulphuric acid (in the ratio 0.3:1.7 respectively). The mixture of chloroform, methanol and sulphuric acid in each sample was flushed with nitrogen gas and tightly sealed with Teflon lined caps and vortexed (vortex mixer) and sonicated (sonication ice bath) for 12 minutes. Samples were subsequently heated to 100°C for 30 minutes in an oven. Thereafter, samples were allowed to stand and cool to room temperature, followed by an addition of 1ml Ultrapure (milliQ) water to each sample. After the addition of water, the solute was centrifuged to separate fatty acid methyl esters and non-fatty acid material. The upper layer (containing non-fatty acid material) of the stratified samples was removed and discarded. The remaining layer of fatty acid methyl esters were dried with sodium sulphate, dissolved in hexane and analysed on a gas chromatograph (GC) at the Central Analytical Facility (CAF), University of Stellenbosch, South Africa. Analysis of fatty acids was completed on an Agilent 6890N GC equipped with a ZB-SemiVolatiles Guardian (30 m, 0.25 mm ID, 0.25 μm film thickness) coupled to an Agilent technologies inert XL EI/CI Mass Selective Detector (5975B, Agilent technologies Inc., Palo Alto, CA). Helium was used as the carrier gas at a flow rate of 1 ml/min. To ensure adequate separation of different fatty acid methyl esters, injector temperature of the gas chromatograph was maintained at 250°C. The oven temperature was programmed as follows: 100°C for 5 min; and then ramped up to 180°C at a rate of 5°C/min and held for 5min and finally ramped up to 330°C at a rate of 8°C/min and held for 5 min.

### Analyses

Owing to the different physiological and energetic demands of male and female bats [46,47], we ran separate analyses for males and females. All statistical and Bayesian analyses were done using R (version 3.5.0) [48].

#### Does *Myotis tricolor* have a wider niche width than *Miniopterus natalensis*?

The isotopic niche width of *M*. *tricolor* and *Miniopterus natalensis* were calculated using SIBER (Stable Isotope Bayesian Ellipses in R version 26 3.3.0; [49]). Bayesian ellipses (generally unbiased with respect to sample size [1,2]) were drawn using SIBER. The niche widths of different *M*. *tricolor* and *Miniopterus natalensis* were represented by the areas within the ellipses and expressed as sample-size corrected standard ellipse areas (SEAc). While small sample sizes (n < 10) result in an underestimation of the population total area (TA), this does not result in a bias in estimates of SEAc [49,50].

Ellipses (generated in SIBER) were also used to compare the fatty acid niche widths of the two-bat species. For the fatty acid data, we initially used non-metric multidimensional scaling (nMDS) of the untransformed fatty acid profiles. We then used the x and y coordinates generated from the nMDS of fatty acids of each individual bat to run SIBER analysis. The areas of the ellipses represented the calculated fatty acid niche widths. Using fatty acids in niche analysis in this way has been successfully used in other studies [44,51]. Values of 95% credibility intervals and Bayesian posterior probabilities (P < 0.05) were used to test for significant differences between species for each location.

#### Proportions of aquatic versus terrestrial insects in the diets of *Myotis tricolor* and *Miniopterus natalensis*

Visual inspection of faeces was used to assess the contributions of different insects to the diet of the two bat species. Pellets from individual bats were placed in a Petri dish and soaked in 90% ethanol, subsequently teased apart with some dissecting pins and forceps under a dissecting microscope [52]. Insect remains were identified to the lowest taxonomic key possible (mostly order) using appropriate regional keys. The percent volume of each insect order was estimated following Whitaker et al. [52]. We deemed the visual analysis to be useful in comparing our study with published findings on the diets of *M*. *tricolor* and *Miniopterus natalensis*.

To further determine the contribution of terrestrial and aquatic insects to the diets of *M*. *tricolor* and *Miniopterus natalensis*, we used the package MixSIAR [53]. This mixing model is based on a series of equations that utilize principles of Bayesian mathematics to determine the proportional contributions of different food sources [54,55]. To run mixing models, trophic discriminations of 1.47 ‰ (SD = 1.51) for δ^15^N and 0.11 ‰ (SD = 0.80) for δ^13^C were applied [16]. Although the discrimination factors are more conservative than those often cited in food web studies (3.4‰ for nitrogen and 1‰ for carbon isotopes), the smaller values correspond to average values extracted from literature specific to stable isotopes of faeces [16,56]. To determine the insects to include in each mixing model, we initially plotted source isotope values against consumer isotope values and discarded sources with very distant isotope values from the bats (Phillips et al. [57]; see S1 to S6 Tables t in supporting information for details of insect taxa included in mixing models). Flying insects destined for dietary analysis were sorted into orders. Potential prey of *M*. *tricolor* and *Miniopterus natalensis* were categorised as having a terrestrial or an aquatic origin. The term ‘‘aquatic prey” was used to describe taxa with aquatic larval and terrestrial adult stages [58]. Additionally, water striders (Gerridae), whirligig beetles (Gyrinidae), water boatmen (Corixidae), Backswimmers (Notonectidae) and water measurers (Hydrometridae) were collected as aquatic prey to represent prey that can only be caught by trawling. These groups, are known to seldom fly [59–61], usually spending most of their lives on the surface of the water and the remainder under water as larvae [62–64]. The term “terrestrial prey” was used for taxa having larvae that develop in the terrestrial ecosystem. Because some insects, owing to their size, cannot be consumed by bats it was necessary to exclude these from the analysis. For instance, insects with body lengths greater than 20mm are too large for both species to consume [65,66] and were excluded from further analysis. Conversely, insects (most of which were terrestrial) with body lengths less than 2mm are too minute to be detected by the echolocation systems of these two species and were also excluded from further analysis [66].

This method ensured that each model was reduced to a three-endpoint mixing model i.e. three broad categories of insects consumed (terrestrial insects, aquatic insects, and trawled insects). Simplifying mixing models to these three food sources allowed for accurate contribution of insects with two stable isotopes [67,68]. Three source mixing models ensure that the models are robust, allowing for feasible solutions [68].

We also ran fatty acid mixing models using fatty acid data from bat faeces (S5 Table). Due to the potential high variability in fatty acids of invertebrate prey [67], we indirectly determined the contributions of terrestrial and aquatic insects using the terrestrial plants and benthic algae (end members) upon which terrestrial and aquatic insects feed. Insect fatty acid values are highly variable in polyphagous terrestrial insects [69]. It was therefore necessary to obtain the proportions of fatty acids in terrestrial (represented by terrestrial plants) and aquatic (represented by benthic algae including epiphyton, epipelon, epilithon) food sources of insect prey. Running mixing models for predators by only including primary producers, rather than insect and other middle trophic level prey allows one to determine what the baseline sources of food are for a consumer’s diet. This allows one to eliminate much variability from the models that would arise as a result of using the actual prey [44,70,71]. To run the mixing models, we used a discrimination factor of ‘zero’ in all our models [72,73].

We quantified the relative contributions of aquatic and terrestrial food to the diets of the two bat species using the ratio of docosahexaenoic acid (DHA; aquatic marker) to Linoleic acid (LIN; terrestrial marker), hereafter referred to as DHA/LIN. This ratio has been proposed as a marker for tracing aquatic and terrestrial contributions in the faecal diets of carnivorous mammals and bat species [17,74]. High DHA: LIN ratios generally indicate higher consumption of aquatic sources relative to terrestrial sources and vice versa.

## Results

### Niche widths of *Miniopterus natalensis* and *Myotis tricolor*

**Fig 2:**
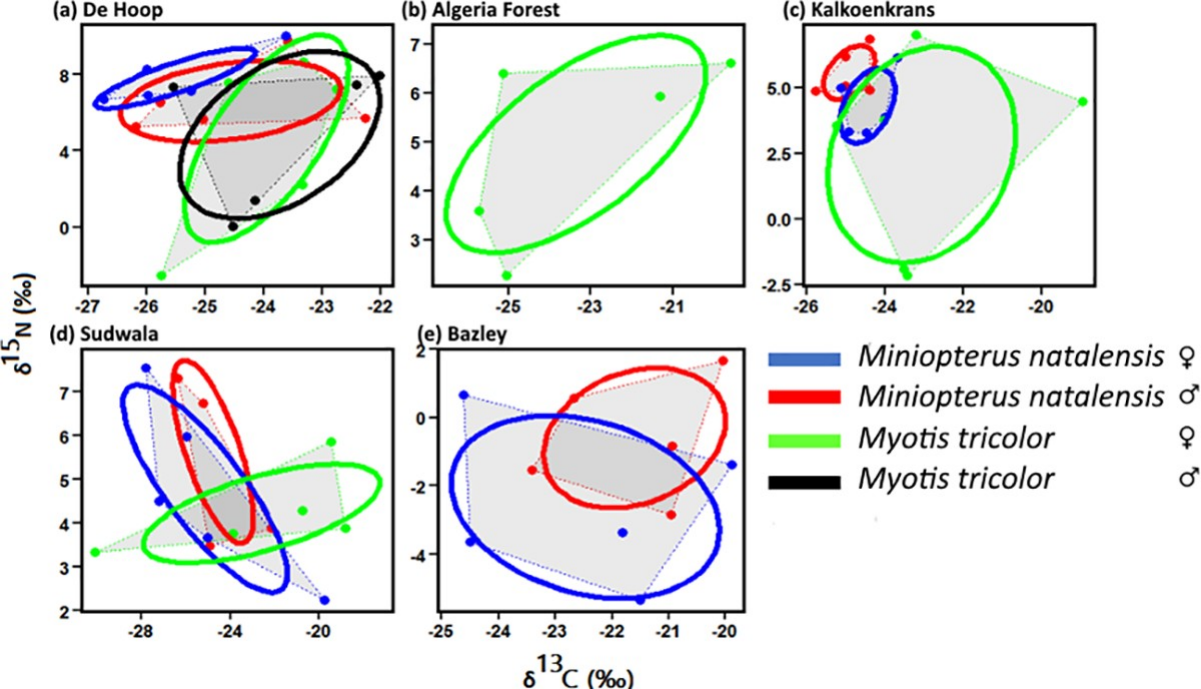
Feeding niche sizes of two sympatric bat species (*Myotis tricolor* and *Miniopterus natalensis;* all samples n = 5 bats), by site, calculated using isotopic (δ^13^C and δ^15^N, ‰). Graphs a, b and c represent sites near large water bodies and graphs d and e represent sites that are much further from water bodies or are close to water bodies with riffle flow. The plots were created using SIBER. Note the change in the isotope axes scales among sites. Area shaded in grey shows area covered by convex hulls.

**Fig 3:**
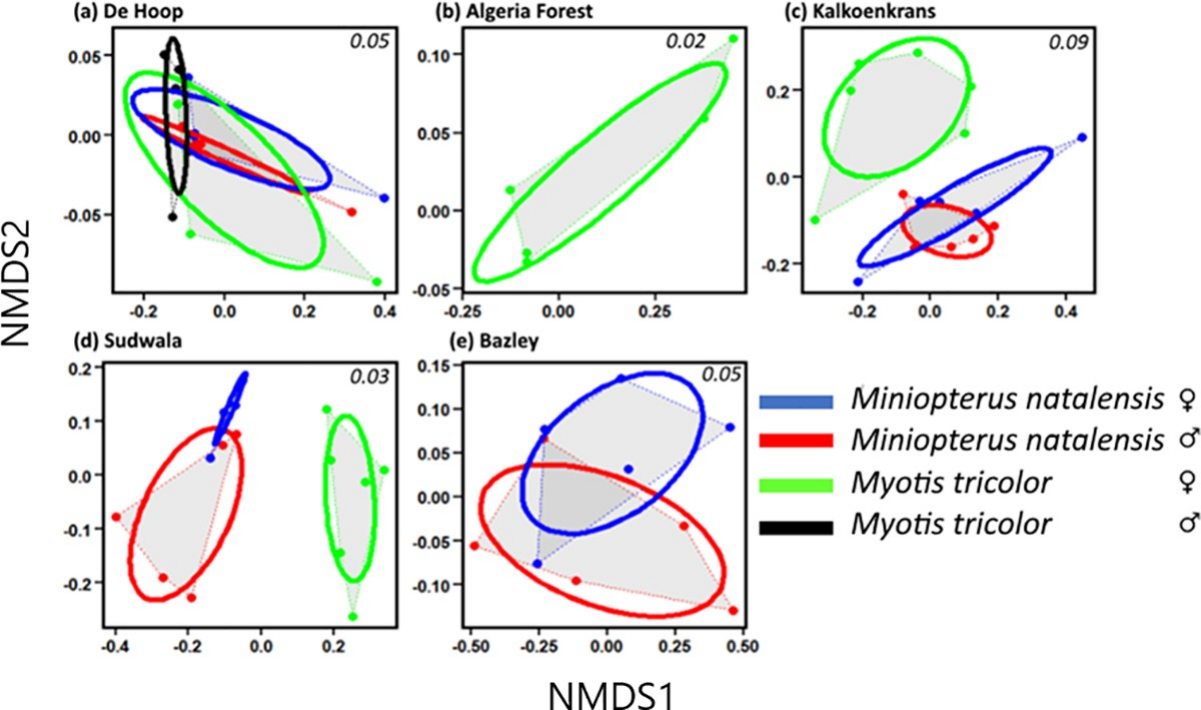
Feeding niche sizes of two sympatric bat species (*Myotis tricolor* and *Miniopterus natalensis*; all samples n = 5 bats), by site, calculated using total fatty acids (%TFA). The plots were created using SIBER. Values in the top right corners of each plot are stress values from non-metric multidimensional scaling. Area shaded in grey shows area covered by convex hulls.

**Fig 4:**
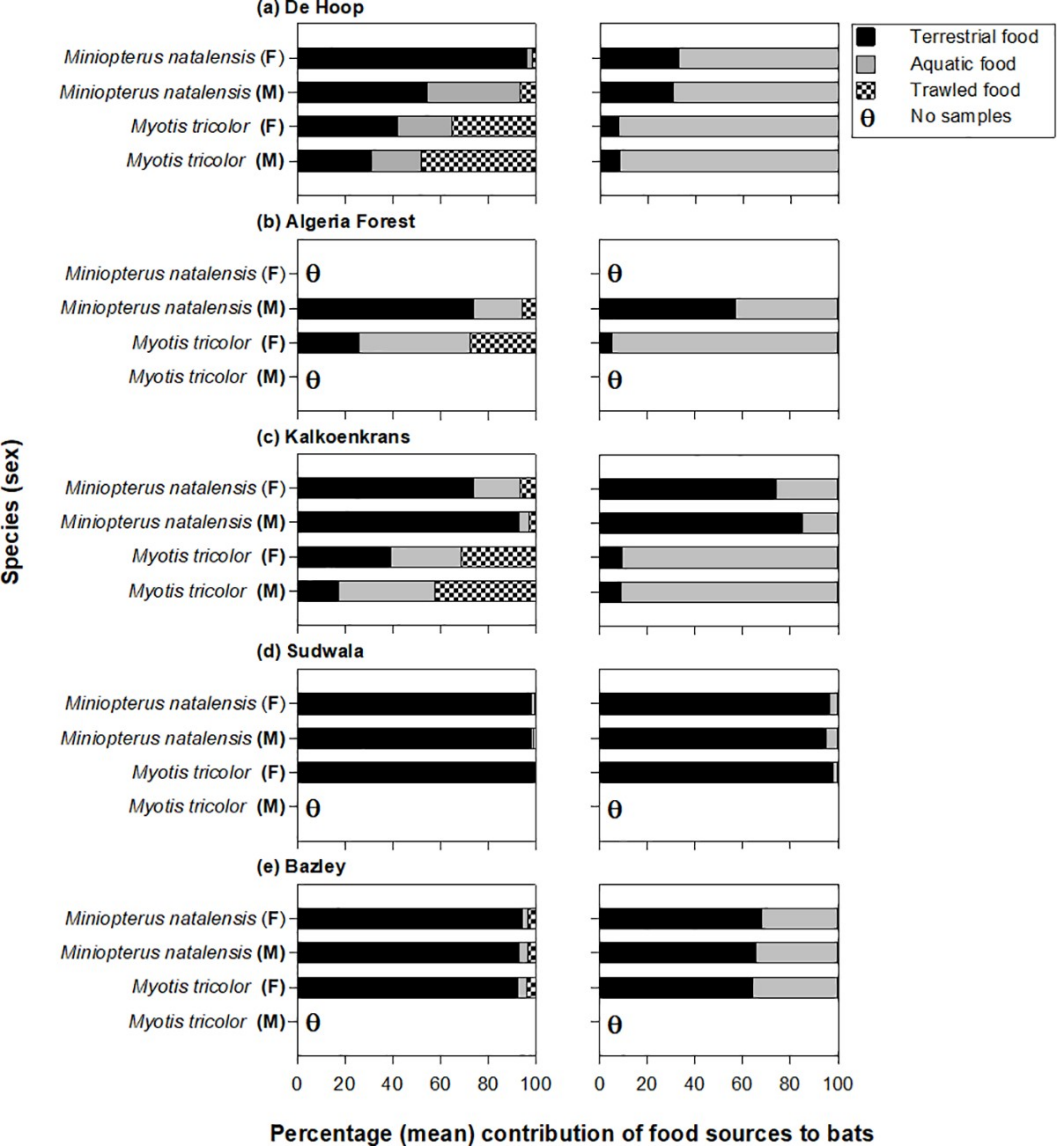
Percentage (mean) aquatic and terrestrial contributions to two sympatric bat species (*Miniopterus natalensis* and *Myotis tricolor*). Stable isotope mixing models (*left panels*) are based on terrestrial, aquatic insects and insects that can only be caught via trawling. Fatty acid mixing models (*right panels*) are based on aquatic and terrestrial consumers. “M” denotes Male and “F” denotes Female. Most samples sizes were n = 3–5bats; where n < 3 bats all mixing models were run using ‘process’ option in MixSIAR.

Dietary niche widths based on isotopes were greater in *Myotis tricolor* than *Miniopterus natalensis* (Fig 2; Table 2). Niche widths of *M*. *tricolor* and *Miniopterus natalensis* based on fatty acids also showed similar patterns. The fatty acid niche widths for *Miniopterus natalensis* were not only narrower than those for *M*. *tricolor* (Fig 3, Table 3), but in some cases were also completely separated from those for *M*. *tricolor* with no overlap between them at Kalkoenkrans and Sudwala (Fig 3C and 3D). At some sites we collected fewer than 5 individuals of *M*. *tricolor* which was not enough to carry out SIBER analysis, hence there are no data for genders and species at Algeria Forestry Station and Bazley Beach (Fig 2B & 2E). In most instances, females had larger niche widths than their male counterparts (Figs 2 and 3 and Table 2). The only exceptions to our prediction were at Sudwala and Bazley, where the niche widths of the two species based on the two markers were similar at the two sites (Figs 2D, 3D, 4D & 4E; Table 2).

**Table 2:** SIBER generated stable isotope and fatty acid niche widths (corrected standard ellipse areas; SEA_c_) of two bat species (*Myotis tricolor* and *Miniopterus natalensis*) at sites in South Africa. SIBER was only run where n = 5 bats. Superscript letters show significant differences (statistical differences were estimated from credibility intervals of posterior distributions).

Site		Isotopic niche size	Fatty acid niche size
n = 5 for all samples			
**De Hoop**			
Near vlei	*Miniopterus natalensis* ♀	3.54^**a**^	0.011^**e**^
	*Miniopterus natalensis* ♂	8.90^**b**^	0.002^**f**^
	*M*. *tricolor ♀*	21.30^**c**^	0.031^**g**^
	*M*. *tricolor* ♂	17.89^**d**^	0.004^**f**^
**Algeria Forest**			
Near large water body	*M*. *tricolor* ♀	16.89	0.030
**Kalkoenkrans**			
Near small river	*Miniopterus natalensis* ♀	2.84^**a**^	0.047^**e**^
	*Miniopterus natalensis* ♂	1.88^**a**^	0.023^**f**^
	*M*. *tricolor ♀*	30.57^**b**^	0.098^**g**^
**Sudwala**			
Near small river	*Miniopterus natalensis* ♀	15.66^**a**^	0.002
With riffles	*Miniopterus natalensis* ♂	8.40^**b**^	0.065
	*M*. *tricolor ♀*	14.70^**c**^	0.032
**Bazley**			
Far from water	*Miniopterus natalensis* ♀	19.21^**a**^	0.084^**e**^
	*Miniopterus natalensis* ♂	10.02^**b**^	0.108^**e**^

**Table 3:** Mean (standard deviation) percent volume of insect orders in the diets of two bat species [*Myotis tricolor* (Mt) and *Miniopterus natalensis (Mn)]* caught at five sampling sites. “n” denotes the number of bats examined. “- “denotes instances when no bat faecal samples were available.

	De Hoop		Algeria Forest		Kalkoenkrans		Sudwala		Bazley	
*n*	5	5	0	1	5	5	5	4	5	2
**Prey**	*Mn*	*Mt*	*Mn*	*Mt*	*Mn*	*Mt*	*Mn*	*Mt*	*Mn*	*Mt*
Coleoptera	45.9(12.9)	10.2(10.6)	-	59	11.0(4.6)	36.9(0.8)	23.3(8.5)	44.8(12.6)	22.2(5.6)	25.7(2.7)
Diptera	3.0(2.9)	0	-	2	42.0 (2.3)	21.6(4.8)	0	0	1.3(2.0)	2.3(4.8)
Ephemeroptera	0	0	-	3	0	0	0	0	0	0
Hemiptera	8.1(2.8)	32.9(12.4)	-	11	19.6 (19.4)	26.1(1.6)	59.1(9.2)	34.9(8.9)	16.9(4.7)	13.4(3.2)
Hymenoptera	15.0(7.8)	35.7(20.6)	-	20	11.8(16.6)	0	5.4(3.7)	12.0(9.0)	4.4 (6.7)	0.3(2.1)
Isoptera	9.5(5.3)	17.1(5.4)	-	0	5.6 (7.9)	0	0	0	35.6 (11.8)	40.5 (10.4)
Lepidoptera	13.5(5.9)	0	-	0	0	0	0	0	0	0
Neuroptera	0	0	-	4	0	14.3(4.0)	0	0		0
Trichoptera	1.4(1.7)	2.6(3.8)	-	1	0	1(3.4)	0	0	0	0
Unknown*	3.6(5.5)	1.4(6.3)	-	0	8.8 (12.5)	0	12.2(6.6)	8.3(2.6)	20.7(1.7)	17.8(6.7)

*Hair fragments and non- arthropod material

### Aquatic versus terrestrial insects in the diet of *Myotis tricolor* and *Miniopterus natalensis*

Stable isotope mixing models revealed that *M*. *tricolor* consumed a suite of terrestrial (Coleoptera, Isoptera, Hemiptera, Hymenoptera; Table 3 & S4 Table) and aquatic insects (e.g. Ephemeroptera, Diptera, Trichoptera; S4 Table). Aquatic insects contributed substantially to *Myotis tricolor* (up to 98%) at De Hoop, Algeria Forestry Station and Kalkoenkrans compared to Bazley and Sudwala (up to 3.8%; Fig 4). At De Hoop, Algeria Forestry Station and Kalkoenkrans, insects that can only be caught via trawling or gleaning (Gerridae, Gyrinidae, Hydrometridae, Corixidae, Notonectidae) made major contributions to the diet of *Myotis tricolor* (up to 45% at De Hoop) and minor contributions to the diet of *Miniopterus natalensis* (Fig 4; S6 Table). Generally, *Miniopterus natalensis* diet was dominated by terrestrial insects (a combination of Coleoptera, Isoptera, Lepidoptera and Hymenoptera; supplementary information) at all sites. However, it also consumed small proportions of insect species (Gyrinidae) that are usually accessible only through trawling, at all sites, with the exception of Sudwala (Fig 4E). Similarly, fatty acid mixing models showed that aquatic insects contributed substantially to *M*. *tricolor and Miniopterus natalensis* at De Hoop, Algeria Forestry Station and Kalkoenkrans (Fig 4) with terrestrial insects being more important to both species at Sudwala and Bazley (64 to 98% between the two sites).

Visual analysis of bat faeces revealed that *Myotis tricolor* and *Miniopterus natalensis* consumed a suite of insects in their diets (Table 3). For instance, Isoptera made moderate contributions to diets except for Bazley where they contributed up to 41% to diets of both *Myotis tricolor* and *Miniopterus natalensis*. *Myotis tricolor* did not consume Lepidoptera, but Lepidopterans made minor contributions to *Miniopterus natalensis* (up to 13.5%). Similarly, Neuroptera made little contribution to consumers at most sampling stations except at Kalkoenkrans (Table 3).

**Fig 5:**
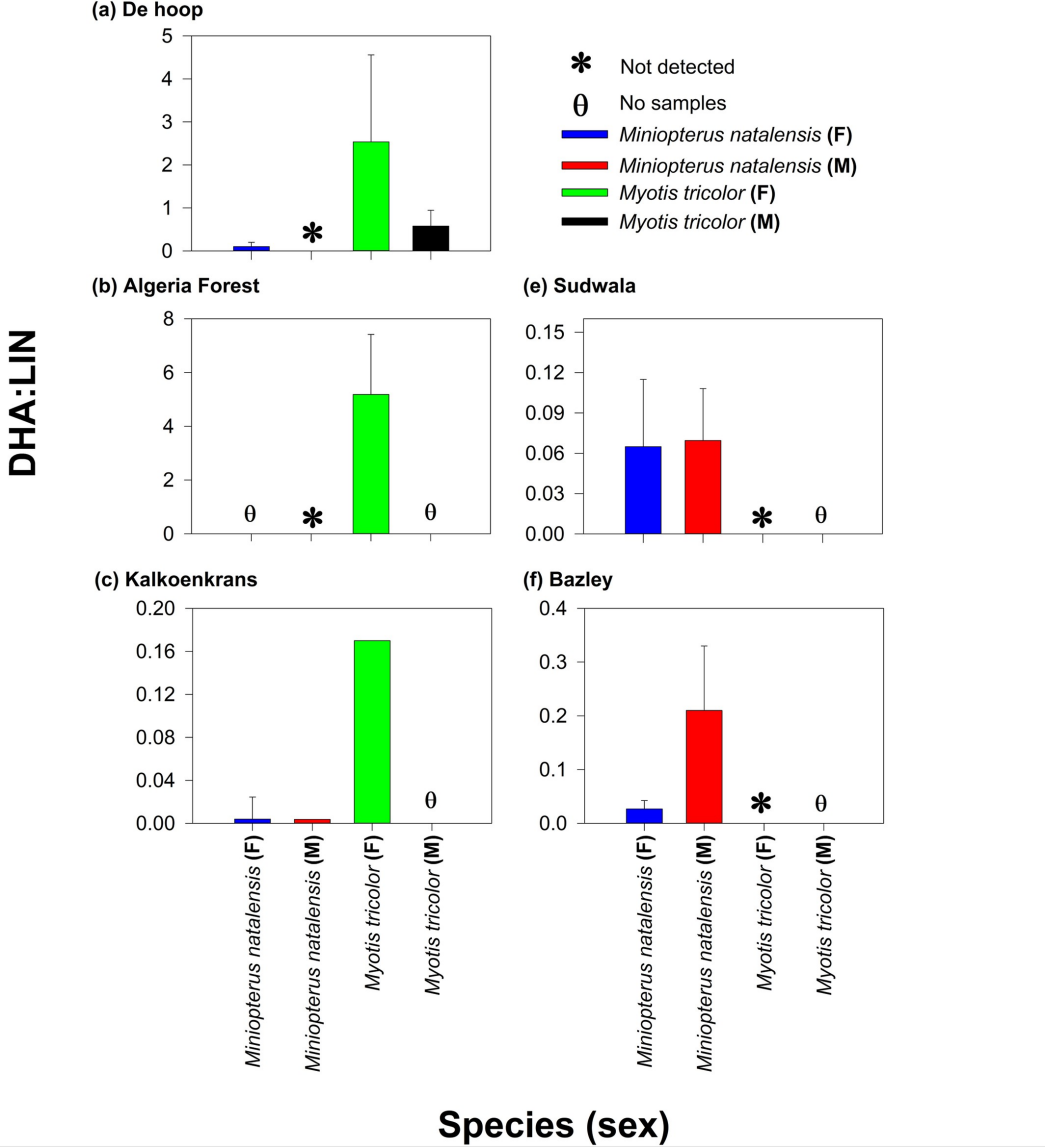
Mean (±SD) of DHA/LIN fatty acid ratios in bats (*Miniopterus natalensis* and *Myotis tricolor*) from five sampling sites in March 2017 and October 2017. Note the change in the DHA/LIN axis among sites. “M” denotes Male and “F” denotes Female. All samples sizes were n = 3–5bats.

DHA:LIN ratios were higher in *M*. *tricolor* than in *Miniopterus natalensis* at all sites (Fig 5). *Myotis tricolor* at De Hoop, Algeria Forestry Station, Kalkoenkrans assimilated the greatest proportions of material originating from aquatic insects. In some instances, the fatty acid ratio DHA/LIN was zero (Fig 5) because some faecal samples had undetectable DHA.

## Discussion

The three different methods of assessing diet used in this study indicated that the niche width of *M*. *tricolor* was generally greater than that of the obligate aerial hawker (*Miniopterus natalensis)*. It appears that the wider niche of *Myotis tricolor at* some sites is, at least in part due, to its greater consumption of aquatic prey, as evidenced by the importance of aquatic insects to its diet (Fig 4). Furthermore, components of the aquatic prey consumed by *M*. *tricolor* are usually only accessible by trawling or gleaning from the surface of water. However, *Miniopterus natalensis* also consumed aquatic insects which included insects accessible by trawling or gleaning, albeit the latter in much smaller proportions than *M*. *tricolor*. At some sites (e.g. Sudwala) both species consumed more terrestrial insects than aquatic insects (Fig 4). Taken together these results suggest much overlap in the diet of both species and much flexibility, at least in the types of insects they consume, allowing them to take advantage of mass emergence of insect prey. However, an important difference is that insects usually only accessible by trawling or gleaning were more prevalent in the diet of *M*. *tricolor*. This may be indicative of differences in the way they forage; *M*. *tricolor* may have a greater preference than *Miniopterus natalensis* for foraging over water and closer to the water surface, or it may also be indicative of trawling in *M*. *tricolor*. Nevertheless, these results suggest that spatial segregation and/or flexibility in foraging mode may sufficiently differentiate the niches of these two species allowing them to co-exist.

The ecomorphological characteristics of *M*. *tricolor* may support its ability to use a dual foraging strategy. *Myotis tricolor* has an average wing loading (8.2 Nm^-2^) and an average aspect ratio (6.2) with short duration, broadband echolocation calls [34]. The morphology of *M*. *tricolor* is typical of many trawling bats [34,75] from other regions of the world (See Table 4). Moreover, their short duration calls are an adaptation for hunting close to substrate because it allows the bat to avoid pulse-echo overlap that would result in the echo arriving at the bats ear when the ear is at its least sensitive. Myotid bats, and other low duty cycle bats, prevent self-deafening by desensitizing their ears as they emit their very loud echolocation pulses [28]. The wing morphology [6], body size [6] and the general occurrence of *M*. *tricolor* over water bodies [11] suggests that this species may be a generalist that utilises more than one foraging strategy; i.e. trawling or gleaning and aerial hawking. Anecdotally, species of *M*. *tricolor* have been caught with wet feet and wet tail membranes (DS Jacobs, personal observations) providing further support for the notion that this species may trawl for insects.

**Table 4:** Published data documenting morphology of typical trawling *Myotis* bats. All trawlers are characterised by large tail and big feet (Fenton & Bogdanowicz 2002). “*” denotes no available data.

Taxa	*M*. *tricolor*	*M*. *daubentonii*	*M*. *dasycneme*	*M*. *capaccinii*
Mass (g)	8–13.4	7–10.2	7–12.4	7–11.8
Tail (mm)	44–51	27–48	29–49	30–49
Feet (mm)	12–14.4	*	*	13.4
Wing loading (Nm^-2^)	8.2	7	6.8	6.5
Aspect ratio	6.2	6.3	10.4	10.5
Forearm (mm)	47–55	33–42	43–49	38–44
Sample size range (n)	3–83	25–93	38	37–198
References	[This study, 1,2]	[91,92]	[91,93]	[91,94,95]

We found the contribution of aquatic versus terrestrial insects to differ among sites, with greatest contributions of aquatic insects (including trawled/gleaned insects) occurring at sites close to large bodies of water with low flow. Specifically, De Hoop (on the banks of the large De Hoop Vlei; seasonal or intermittent lake), Algeria Forestry Station (on the banks of the Rondegat River with calm pools) and Kalkoenkrans (near a small slow flowing river) were sites close to water bodies that may have provided suitable habitats for trawling or gleaning and aerial hawking. This may explain why aquatic insects (along with those that can be caught via trawling/gleaning) made substantial contributions to the diet of *M*. *tricolor*. This is plausible considering that trawling bats prefer to hunt over calm water compared to water that has plants (e.g. duckweed and water hyacinth), artificial objects, or turbulent, riffled flow [75–77]. The water flow and narrowness of the streams at some sites (e.g. Sudwala) may have precluded trawling and gleaning because riffles and rapids would make detection of insects by echolocation difficult and aquatic plants would impede the low flight over the water required for these foraging strategies. At Sudwala, the Houtbosloop River ran through the site providing a potential habitat for trawling or gleaning. However, the section of the river took the form of a narrow stream with many riffles [78] and aquatic plants growing in the stream (S Moyo, personal observation), which may have prevented bats from capturing aquatic prey from this site.

Traditionally, *M*. *tricolor* has been considered an aerial hawker mainly dependent on terrestrial insect prey, largely because many of the studies have been based on visual inspection of faecal analysis [79]. However, a combination of visual inspection and alimentary tracers (fatty acids and stable isotopes) revealed that *M*. *tricolor* feeds on a combination of aquatic and terrestrial prey; which may have been missed by reliance on one method. Data obtained from our study sites suggest that aquatic prey contributed more to diets of *M*. *tricolor* occurring near calm lotic/lentic systems (De Hoop and Algeria Forestry Station) relative to sites occurring further from large water bodies and close to rapid water flow. The putative ratio for reliance of bats on terrestrial versus aquatic insects was higher in diets of *M*. *tricolor* closer to large water bodies relative to those away from large water bodies (Fig 5), suggesting that the dependence of bats on different food prey changed spatially. The prevalence of DHA/LIN in *M*. *tricolor* (0.25 to 5 across all sites) is typical of other bats known to trawl (DHA: LIN = 0.75 total fatty acids in *Myotis daubentonii* versus 0.25 total fatty acids in *Myotis myotis*; Lam et al. [17] estimated from Fig 4C) providing a line of evidence that *M*. *tricolor* may be a facultative trawling species.

Correspondingly, mixing models showed that aquatic and terrestrial insects make disparate contributions to *M*. *tricolor* depending on their proximity to different prey sources; with aquatic insects and terrestrial insects all contributing to the diets of this species. The only completely aquatic taxon observed in the diet of bats were the families, Notonectidae, Hydrometridae, Gerridae and Gyrinidae that contributed large proportions (≈ 45%) to *M*. *tricolor* at sites that were close to water. Completely aquatic taxa are also the most consumed arthropod in trawling bat species in north-west Israel (*Myotis capaccinii;* [80]) and Japan (*Myotis daubentonii;* [81]). Furthermore, *M*. *tricolor* closer to calm water had larger niche widths (SEAc) than *Miniopterus natalensis* (an obligate hawker) suggesting differences in foraging behaviours. Disparate feeding niche widths are typical of species utilising different food resources [50]. This is tenable, especially considering that faecal analysis of eight species of bats revealed that species utilising similar foraging strategies had similar dietary compositions and ultimately occupied similar dietary niches while gleaners and aerial hawkers had disparate dietary compositions [22].

Variations in foraging strategies within the study areas, reinforced the foraging flexibility of *M*. *tricolor* (in support of the expectation that the dominant food resources utilized by bat species vary among sites mainly as a function of prey availability). However, some differences occurred between the datasets produced using different methods. This reflects inherent biases and strengths of the different methods used to analyze faeces to determine animal diets. Alimentary tracers provided greater resolution of dietary components allowing the identification of aquatic insects that required trawling for capture (S2 Table). These were missed in the visual inspection of faeces only. Similarly, different tracers also showed more or less facility. For example, fatty acids and stable isotopes showed different contributions of aquatic versus terrestrial prey in the diets of bats (Fig 4A), reflecting the strength of using a combination of biochemical tracers (stable isotopes and fatty acids) with visual analysis to estimate the proportions of different foods assimilated by bats.

At sites close to the water (De Hoop, Kalkoenkrans and Algeria forest), *M*. *tricolor* probably favoured lentic and lotic systems, where this species benefits from the abundance of aquatic insects emerging from the larger water bodies. The new dietary information on *M*. *tricolor* produced here suggests the foraging plasticity of this species allows it to take advantage of changes in insect abundance. At sites and different times where aquatic insect abundance may be low *M*. *tricolor* is able to switch to aerial hawking and take advantage of terrestrial insects. For example, at sites further from water the diets of *Miniopterus natalensis* and *M*. *tricolor* were similar with terrestrial insects making large contributions to the diets of these species. At one site (Bazley) there was a mass emergence of Isoptera during one of the sampling nights and it appears that both species took advantage of this abundant and easy to catch prey as is evidenced by the high proportion of Isoptera in the diets of bats from this site with relatively low proportions of aquatic insects. This suggest that the species may shift to a particular foraging strategy based on food availability. This is common in other *Myotis* bat species that are known to use more than one foraging strategy depending on prey availability [7].

Our results are supported by studies on other trawling *Myotis* species as reflected by a high proportion of arthropods with complete or semi aquatic life cycles. Specifically, high proportions of Gerridae and Gyrinidae that are completely aquatic and abound in lentic and lotic systems throughout their life cycles [82], have been shown to be typical for other trawling *Myotis* species like *M*. *capaccinii* [83]. Overall dietary results across the group of trawling *Myotis* species are congruent to our findings, with many semi aquatic insects (Ephemeroptera, Plecoptera and Simuliidae) and fully aquatic (Gerridae, Gyrinidae) as main prey groups as was the case for *M*. *tricolor* in our study. Among trawling *Myotis*, there are few species known to prey upon fish e.g. in Europe, *Myotis capaccinii* and *Myotis daubentonii* are known to trawl for fish [83]. Considering the regular presence of fish in several species of bats that forage close to water surfaces, it seems plausible that the *M*. *tricolor* may very well trawl for fish too. However, our data (using faecal) analysis did not reveal presence of fish scales across several sites, so it is unlikely that *M*. *tricolor* trawls for fishes, at least at the sites we sampled, despite fish being observed at both De Hoop and Algeria Forestry Station. Nevertheless, given the foraging plasticity of this species demonstrated here it cannot be excluded without more extensive and intensive sampling.

The visual analysis of bat faeces revealed the ingestion of a variety of food resources by *M*. *tricolor and Miniopterus natalensis*. However, contrary to the results from alimentary tracers, we found little evidence of soft-bodied aquatic insects like trichopterans and ephemeropterans through visual inspection of faeces. This is striking especially considering that we identified trichopterans and ephemeropterans in the diet of *M*. *tricolor* using alimentary tracers. These insects abound in freshwater and terrestrial communities, and they are usually found in the diet of other trawling bats as well [83]. The discrepancy between dietary analyses based on arthropod fragments in faeces and tracers is probably due to under-representation of these soft-bodied aquatic insects among insect fragments in faeces [84]. Nevertheless, analyses of insect fragments in faeces provided a useful guideline for the selection of appropriate food resources to be analyzed using the biochemical techniques. This illustrates the advantage of combining traditional analyses with other methods for dietary assessment.

The occurrence of insect species that could usually only be obtained by a bat through trawling or gleaning in an obligate aerial hawker like *Miniopterus natalensis* can be explained by species like Gyrinidae, Corixidae and Hydrometra that live underwater, but approach the surface to breathe, and occasionally fly [82] to disperse to other water systems. It is unknown whether these insects were captured by *Miniopterus natalensis* while flying or perhaps incidentally ingested while bats drank from the surface when these insects come to the water surface to breathe. Other obligate aerial hawking bats (e.g. *Tadarida brasilensis*) have been recorded to prey on Corixidae in flight [85] and *Miniopterus natalensis* may do so too. However, given the low proportions of aquatic insects that require trawling or gleaning in the diet of *Miniopterus natalensis* relative to that in the diet of *M*. *tricolor*, it is likely that these insects were incidentally ingested by *Miniopterus natalensis* as it drank water. This is supported by the trace proportions of such insects in the diets of both *M*. *tricolor* and *Miniopterus natalensis* at Bazley where opportunities for trawling or gleaning were not available.

The differences in feeding niches between male and female bats may be attributable to the different energetic demands of each sex (e.g. hormone levels, lactation, water balance, fat to muscle tissue ratio, body size) [86,87] which may in turn affect the amount of food they process or require. For instance, females of long tailed bats (*Chalinolobus tuberculatus*) usually have longer feeding bouts than their male counterparts owing to their higher energetic demands (especially lactating females; [88]).

We were not always able to collect both genders of both *M*. *tricolor* and *Miniopterus natalensis* at some sites, so some caution is necessary when interpreting our results. Additionally, our study lacked a replication of sites. However, this may have had only a minor effect on our interpretation because we used complimentary methods [17] and limited our inferences to the sites we sampled. Further studies are needed to further assess the foraging ecology of *M*. *tricolor* in other parts of Africa where males and females occur together in abundance. Additionally, the importance of certain fatty acids to males and females and whether the proportion of these fatty acids in their diets is associated with different physiological needs of males and females would help elucidate the reasons behind dietary differences between males and females.

In all our analyses of fatty acids, we are cognisant that our approach is predicated on several assumptions: (1) that benthic algae and C_3_ terrestrial plants are the two main sources for primary productivity in the system; (2) that fatty acids do not vary greatly with changing trophic levels, between terrestrial plants and algae, or among consumers; (3) that measured fatty acid profiles in bat faeces are representative of the diet of the bats. We are however confident that our models are representative of the contribution of terrestrial and aquatic insects to bat diets. Additionally, while fatty acids may vary depending on the tissues sampled, the applicability of using fatty acids to study diet in faeces has been validated by other researchers [17].

### General conclusions and considerations

Our findings (based on alimentary tracers) highlight the need for a re-assessment of the *M*. *tricolor* guild designation as just an aerial hawker. Our data indicate that *M*. *tricolor* at several sites assimilated a combination of volant insects (terrestrial and aquatic) and insects that swim on the surface of the water (obtainable by trawling, gleaning or aerial hawking), with a heavy reliance on aquatic food. This result is consistent with a plethora of studies documenting the foraging ecology of known trawlers (e.g., *Myotis daubentonii*; [17]), where fatty acid metrics showed high proportions of fatty acids (DHA and LIN) typical of trawlers. Furthermore, while *M*. *tricolor* is often associated with the aerial hawking guild we often caught many *M*. *tricolor* with wet feet and uropatagia at sites close to the water. While there may be several potential explanations for this observation e.g. bats drinking water from the water surface, other *Myotis* spp. with similar large feet and uropatagium [7,89] are known to typically hunt low over water, and often seize prey from the water’s surface using their feet and uropatagia. We cannot rule out gleaning as a potential strategy employed by *Myotis tricolor* and further studies may generate interesting findings on this question. However, considering that a study on the foraging behaviour of *Myotis tricolor* suggests it does not glean [34] it is highly likely that it may be a trawler analogous to many *Myotis* species. Nonetheless, our findings show that *Myotis tricolor* is a generalist forager.

Based on these different lines of evidence, we recommend that additional tracer-based studies (including barcoding methods) be conducted on bats using blood, fur and muscle tissue to characterise assimilated diets over several months, to confirm the relative reliance on different foraging modes and prey items over space and time. Our results indicate the utility of alimentary tracers in understanding the foraging behaviour of cryptic species but emphasizes that a combination of methods is likely to yield greater insights into the diets of animals than any one of them on its own.

## Supporting information

S1 Table Isotopic values for De Hoop Nature Reserve and Algeria Forestry Station. Summary of isotopic values (‰; Mean ± SD) for *Miniopterus natalensis*, *Myotis tricolor* and several orders of insect taxa at De Hoop Nature Reserve and Algeria Forestry Station.
(DOCX)

S2 Table Isotopic values for Sudwala and Kalkoenkrans. Summary of isotopic values (‰; Mean ± SD) for *Miniopterus natalensis*, *Myotis tricolor* and several orders of insect taxa at Sudwala and Kalkoenkrans.
(DOCX)

S3 Table Isotopic values for Bazley Beach. Summary of isotopic values (‰; Mean ± SD) for *Miniopterus natalensis*, *Myotis tricolor* and several orders of insect taxa at Bazley Beach.
(DOCX)

S4 Table Mixing models. Arthropod taxa included in the mixing models for the two bat species (*Myotis tricolor* and *Miniopterus natalensis*) at five sampling stations (see S3 Table for summary of isotope values).
(DOCX)

S5 Table Fatty acid values. Summary of fatty acid values (% Total fatty acids (TFA); Mean ± SD) of bats (*Miniopterus natalensis* and *Myotis tricolor*), terrestrial plants and benthic algae.
(DOCX)

S6 Table Results of Bayesian mixing models. Mean percentage contributions (credibility intervals displayed in parentheses) of food sources from Bayesian mixing models (MixSIAR). Stable isotope mixing models were based on insect prey while fatty acid mixing models were based on benthic algae and terrestrial plants as proxies for aquatic and terrestrial insects. See S2 Table for the arthropod taxa placed in the three categories: aquatic, terrestrial and trawled.
(DOCX)
