# Supplementary material for: Faecal analyses and alimentary tracers reveal the foraging ecology of two sympatric bats
Source: PLoS One. 2020 Jan 16;15(1):e0227743. doi: 10.1371/journal.pone.0227743 (PMC6964858; doi:10.1371/journal.pone.0227743)
Supplement: S1 Table — Summary of isotopic values (‰; Mean ± SD) for Miniopterus natalensis, Myotis tricolor and several orders of insect taxa at De Hoop Nature Reserve and Algeria Forestry Station. (DOCX) [file pone.0227743.s001.docx]

**S1 Table. Isotopic values for De Hoop Nature Reserve and Algeria Forestry Station.** Summary of isotopic values (‰; Mean ± SD) for Miniopterus natalensis, Myotis tricolor and several orders of insect taxa at De Hoop Nature Reserve and Algeria Forestry.

| Taxa | δ^15^N | SD |  | δ^13^C | SD |
| --- | --- | --- | --- | --- | --- |
|  |  |  |  |  |  |
| **De Hoop** |  |  |  |  |  |
| *Miniopterus natalensis (♀)* | 6.60 | 1.83 |  | -24.57 | 1.63 |
| *Miniopterus natalensis (♂)* | 7.81 | 1.37 |  | -25.51 | 1.18 |
| *Myotis tricolor (♀)* | 4.63 | 4.71 |  | -23.96 | 1.21 |
| *Myotis tricolor (♂)* | 4.84 | 3.80 |  | -23.73 | 1.48 |
| Gyrinidae | 6.91 | 0.64 |  | -25.86 | 0.16 |
| Gerridae | 7.22 | 1.55 |  | -24.65 | 1.78 |
| Notonectidae | 6.73 | 0.23 |  | -26.48 | 0.45 |
| Ephemeroptera | 8.01 | 0.84 |  | -25.22 | 0.83 |
| Coleoptera | 7.17 | 0.63 |  | -24.07 | 3.77 |
| Diptera | 9.95 | 0.51 |  | -21.23 | 1.01 |
| Hemiptera | 3.25 | 0.40 |  | -23.03 | 0.15 |
| Isoptera | 2.66 | 0.20 |  | -26.10 | 0.11 |
| Lepidoptera | 8.49 | 1.41 |  | -24.51 | 1.61 |
| Hymenoptera | 4.12 | 1.25 |  | -23.61 | 0.24 |
|  |  |  |  |  |  |
| **Algeria** |  |  |  |  |  |
| *Miniopterus natalensis (♀)* | -26.32 | 0.18 |  | 8.03 | 0.80 |
| *Myotis tricolor (♀)* | -23.36 | 2.72 |  | 4.96 | 1.92 |
| Gyrinidae | -24.97 | 0.71 |  | 6.20 | 0.60 |
| Gerridae | -25.99 | 1.04 |  | 4.64 | 0.92 |
| Notonectidae | -24.14 | 0.28 |  | 5.24 | 0.25 |
| Ephemeroptera | -26.38 | 0.40 |  | 4.84 | 0.99 |
| Trichoptera | -27.05 | 0.58 |  | 4.41 | 0.36 |
| Coleoptera | -25.93 | 0.09 |  | 7.81 | 2.46 |
| Simuliidae | -24.35 | 0.21 |  | 3.80 | 0.04 |
| Diptera | -24.59 | 1.00 |  | 5.88 | 3.38 |
| Hemiptera | -23.66 | 0.10 |  | 3.61 | 0.14 |
| Lepidoptera | -25.67 | 1.20 |  | 11.36 | 3.93 |
| Hymenoptera | -27.65 | 0.77 |  | 8.74 | 2.65 |
| Neuroptera | -16.30 | 6.94 |  | 4.59 | 1.82 |
