# Supplementary material for: Faecal analyses and alimentary tracers reveal the foraging ecology of two sympatric bats
Source: PLoS One. 2020 Jan 16;15(1):e0227743. doi: 10.1371/journal.pone.0227743 (PMC6964858; doi:10.1371/journal.pone.0227743)
Supplement: S2 Table — Summary of isotopic values (‰; Mean ± SD) for Miniopterus natalensis, Myotis tricolor and several orders of insect taxa at Sudwala and Kalkoenkrans. (DOCX) [file pone.0227743.s002.docx]

**S2 Table. Isotopic values for Sudwala and Kalkoenkrans.** Summary of isotopic values (‰; Mean ± SD) for Miniopterus natalensis, Myotis tricolor and several orders of insect taxa at Sudwala and Kalkoenkrans.

| Taxa |  | δ^15^N | SD |  | δ^13^C | SD |
| --- | --- | --- | --- | --- | --- | --- |
|  | |  |  |  |  |  |
| **Sudwala** | |  |  |  |  |  |
| *Myotis tricolor (♀)* | | 4.23 | 0.97 |  | -22.59 | 4.6 |
| Gyrinidae | | 6.97 | 0.3 |  | -22.48 | 1.31 |
| Gerridae | | 8.14 | 0.45 |  | -22.13 | 0.49 |
| Ephemeroptera | | 4.51 | 0.25 |  | -22.12 | 0.39 |
| Trichoptera | | 6.16 | 0.42 |  | -23.01 | 0.48 |
| Plecoptera | | 7.52 | 0 |  | -21.29 | 0 |
| Simuliidae | | 8.84 | 0 |  | -22.1 | 0 |
| Hymenoptera | | 2.79 | 0.7 |  | -23.69 | 0.24 |
| Coleoptera | | 1.32 | 2.52 |  | -24.74 | 0.24 |
| Hemiptera | | 5.16 | 1 |  | -24.99 | 2.39 |
| Diptera | | 9.96 | 0 |  | -25.06 | 0 |
| Lepidoptera | | 4.64 | 0.14 |  | -31.8 | 0.23 |
|  | |  |  |  |  |  |
| **Kalkoenkrans** | |  |  |  |  |  |
| *Miniopterus natalensis (♀)* | | 5.58 | 0.89 |  | -24.92 | 0.57 |
| *Miniopterus natalensis (♂)* | | 4.32 | 1.23 |  | -24.45 | 0.61 |
| *Myotis tricolor (♀)* | | 2.2 | 3.02 |  | -22.87 | 2.34 |
| *Myotis tricolor (♂)* | | 3.75 | 0 |  | -24.04 | 0 |
| Gyrinidae | | 8.16 | 0.4 |  | -25.8 | 0.32 |
| Gerridae | | 9.26 | 0.2 |  | -24.67 | 0.16 |
| Ephemeroptera | | 7.59 | 0.05 |  | -25.43 | 0.24 |
| Neuroptera | | 4.69 | 0.99 |  | -23.35 | 3.09 |
| Hemiptera | | 5.72 | 2.75 |  | -22.14 | 3.69 |
| Hymenoptera | | 6.76 | 0.09 |  | -27.03 | 0.04 |
| Coleoptera | | 5.13 | 0.91 |  | -27.07 | 0.31 |
| Lepidoptera | | 10.79 | 0.2 |  | -24.81 | 0.29 |
