# Supplementary material for: Faecal analyses and alimentary tracers reveal the foraging ecology of two sympatric bats
Source: PLoS One. 2020 Jan 16;15(1):e0227743. doi: 10.1371/journal.pone.0227743 (PMC6964858; doi:10.1371/journal.pone.0227743)
Supplement: S3 Table — Summary of isotopic values (‰; Mean ± SD) for Miniopterus natalensis, Myotis tricolor and several orders of insect taxa at Bazley Beach. (DOCX) [file pone.0227743.s003.docx]

**S3 Table. Isotopic values for Bazley Beach.** Summary of isotopic values (‰; Mean ± SD) for Miniopterus natalensis, Myotis tricolor and several orders of insect taxa at Bazley Beach.

| Taxa | δ^15^N | SD |  | δ^13^C | SD |
| --- | --- | --- | --- | --- | --- |
|  |  |  |  |  |  |
| **Bazley** |  |  |  |  |  |
| *Myotis tricolor (♀)* | -19.24 | 1.13 |  | 3.97 | 0.43 |
| *Miniopterus natalensis (♂)* | -21.6 | 1.39 |  | -0.59 | 1.77 |
| *Miniopterus natalensis (♀)* | -22.47 | 2.05 |  | -2.61 | 2.32 |
| Coleoptera | -24.57 | 3.13 |  | 4.24 | 0.35 |
| Ephemeroptera | -27.04 | 0.82 |  | 8.97 | 0.25 |
| Gerridae | -23.29 | 0.07 |  | 9.47 | 0.25 |
| Gyrinidae | -23.73 | 0.6 |  | 8.79 | 0.98 |
| Hemiptera | -12.26 | 0.09 |  | 3.9 | 0.53 |
| Hymenoptera | -24.63 | 0.17 |  | 7.18 | 0.09 |
| Isoptera | -18.13 | 0.56 |  | 3.02 | 0.14 |
| Lepidoptera | -30.24 | 0.33 |  | 6.37 | 0.17 |
| Mecoptera | -22.83 | 0 |  | 8.38 | 0 |
| Plecoptera | -23.22 | 1.24 |  | 10.92 | 0.3 |
| Simuliidae | -23.22 | 0.19 |  | 8.13 | 0.16 |
| Trichoptera | -24.47 | 0.24 |  | 9.94 | 0.04 |
