# Supplementary material for: Faecal analyses and alimentary tracers reveal the foraging ecology of two sympatric bats
Source: PLoS One. 2020 Jan 16;15(1):e0227743. doi: 10.1371/journal.pone.0227743 (PMC6964858; doi:10.1371/journal.pone.0227743)
Supplement: S4 Table — Arthropod taxa included in the mixing models for the two bat species (Myotis tricolor and Miniopterus natalensis) at five sampling stations (see S3 Table for summary of isotope values). (DOCX) [file pone.0227743.s004.docx]

**S4 Table. Mixing models.** Arthropod taxa included in the mixing models for the two bat species (Myotis tricolor and Miniopterus natalensis) at five sampling stations (see S3 Table for summary of isotope values).

|  | **Aquatic** | **Terrestrial** | **Trawled** |
| --- | --- | --- | --- |
| **De Hoop** |  |  |  |
| *M.natalensis (♀)* | Ephemeroptera, Simuliidae | Coleoptera, Isoptera, Lepidoptera | Gyrinidae, Gerridae, Corixidae |
| *M.natalensis (♂)* | Ephemeroptera, Simuliidae | Coleoptera, Isoptera, Lepidoptera | Gyrinidae, Gerridae, Corixidae |
| *M.tricolor (♀)* | Ephemeroptera, Simuliidae | Hemiptera, Isoptera, Hymenoptera | Gyrinidae, Gerridae, Corixidae |
| *M.tricolor (♂)* | Ephemeroptera, Simuliidae | Hemiptera, Isoptera, Hymenoptera | Gyrinidae, Gerridae, Corixidae |
|  |  |  |  |
| **Algeria** |  |  |  |
| *M.natalensis (♂)* | Ephemeroptera, Trichoptera | Coleoptera, Hymenoptera, Diptera | Gyrinidae, Gerridae, Corixidae |
| *M.tricolor (♀)* | Ephemeroptera, Trichoptera | Diptera, Hemiptera | Gyrinidae, Gerridae, Corixidae |
|  |  |  |  |
| **Kalkoenkrans** |  |  |  |
| *M.natalensis (♀)* | Ephemeroptera | Hymenoptera, Coleoptera, Hemiptera, Neuroptera | Gyrinidae, Gerridae, Notonectidae |
| *M.natalensis (♂)* | Ephemeroptera | Hymenoptera, Coleoptera, Hemiptera, Neuroptera | Gyrinidae, Gerridae, Notonectidae |
| *M.tricolor (♀)* | Ephemeroptera | Hymenoptera, Coleoptera, Hemiptera, Neuroptera | Gyrinidae, Gerridae, Notonectidae |
|  |  |  |  |
| **Sudwala** |  |  |  |
| *M.natalensis (♀)* | Ephemeroptera, Trichoptera, Plecoptera | Hymenoptera, Coleoptera, Hemiptera | Gyrinidae, Gerridae |
| *M.natalensis (♂)* | Ephemeroptera, Trichoptera, Plecoptera | Hymenoptera, Coleoptera, Hemiptera | Gyrinidae, Gerridae |
| *M.tricolor (♀)* | Ephemeroptera, Trichoptera, Plecoptera | Hymenoptera, Coleoptera, Hemiptera | Gyrinidae, Gerridae |
|  |  |  |  |
| **Bazley** |  |  |  |
| *M.natalensis (♀)* | Ephemeroptera, Plecoptera, Simuliidae, Trichoptera | Coleoptera, Isoptera | Gyrinidae, Gerridae |
| *M.natalensis (♂)* | Ephemeroptera, Plecoptera, Simuliidae, Trichoptera | Coleoptera, Isoptera | Gyrinidae, Gerridae |
| *M.tricolor (♀)* | Ephemeroptera, Plecoptera, Simuliidae, Trichoptera | Coleoptera, Isoptera | Gyrinidae, Gerridae |
