# Supplementary material for: Faecal analyses and alimentary tracers reveal the foraging ecology of two sympatric bats
Source: PLoS One. 2020 Jan 16;15(1):e0227743. doi: 10.1371/journal.pone.0227743 (PMC6964858; doi:10.1371/journal.pone.0227743)
Supplement: S5 Table — Summary of fatty acid values (% Total fatty acids (TFA); Mean ± SD) of bats (Miniopterus natalensis and Myotis tricolor), terrestrial plants and benthic algae. (DOCX) [file pone.0227743.s005.docx]

**S5 Table. Fatty acid values.** Summary of fatty acid values (% Total fatty acids (TFA); Mean ± SD) of bats (Miniopterus natalensis and Myotis tricolor), terrestrial plants and benthic algae.

| Taxa | **18:2ω6** | | **18:3ω3** | | **20:4ω6** | | **20:5ω3** | | **22:6ω3** | |
| --- | --- | --- | --- | --- | --- | --- | --- | --- | --- | --- |
|  | Mean | SD | Mean | SD | Mean | SD | Mean | SD | Mean | SD |
| **De Hoop** |  |  |  |  |  |  |  |  |  |  |
| *Miniopterus natalensis* (♀) | 7.2 | 3.8 | 5.7 | 2.8 | 0.6 | 0.3 | 6.4 | 5.5 | 0.0 | 0.0 |
| *Miniopterus natalensis* (♂) | 3.1 | 3.0 | 2.9 | 2.0 | 0.4 | 0.2 | 6.4 | 1.2 | 0.0 | 0.0 |
| *Myotis tricolor* (♀) | 4.1 | 3.9 | 3.2 | 3.3 | 0.6 | 0.8 | 9.7 | 9.7 | 2.3 | 1.3 |
| *Myotis tricolor* (♂) | 4.7 | 5.2 | 2.8 | 2.3 | 0.4 | 0.4 | 5.6 | 3.1 | 1.5 | 0.4 |
| Benthic algae | 0.1 | 0.1 | 0.8 | 1.3 | 0.4 | 0.4 | 4.1 | 2.7 | 1.2 | 0.8 |
| Terrestrial plant | 5.8 | 3.3 | 16.2 | 17.7 | 0.2 | 0.2 | 0.1 | 0.2 | 0.0 | 0.0 |
|  |  |  |  |  |  |  |  |  |  |  |
| **Algeria** |  |  |  |  |  |  |  |  |  |  |
| *Miniopterus natalensis* (♂) | 0.2 | 0.0 | 4.3 | 0.0 | 0.2 | 0.0 | 19.2 | 0.0 | 0.0 | 0.0 |
| *Myotis tricolor* (♀) | 15.0 | 5.0 | 1.8 | 2.0 | 1.8 | 0.8 | 6.4 | 4.8 | 4.3 | 2.0 |
| *Myotis tricolor* (♂) | 3.7 | 4.6 | 0.6 | 0.8 | 0.1 | 0.1 | 3.4 | 2.0 | 1.9 | 2.3 |
| Benthic algae | 1.1 | 1.4 | 2.0 | 3.4 | 0.7 | 0.5 | 5.5 | 4.3 | 4.1 | 6.1 |
| Terrestrial plant | 0.9 | 1.4 | 1.6 | 0.1 | 0.2 | 0.1 | 0.0 | 0.0 | 0.0 | 0.0 |
|  |  |  |  |  |  |  |  |  |  |  |
| **Sudwala** |  |  |  |  |  |  |  |  |  |  |
| *Miniopterus natalensis* (♂) | 7.4 | 2.7 | 7.8 | 5.4 | 1.4 | 0.8 | 0.3 | 0.3 | 0.7 | 0.6 |
| *Miniopterus natalensis* (♀) | 9.1 | 1.9 | 9.2 | 3.5 | 1.1 | 0.1 | 0.3 | 0.2 | 0.5 | 0.4 |
| *Myotis tricolor* (♀) | 15.0 | 6.5 | 1.6 | 1.4 | 4.3 | 1.8 | 0.6 | 0.0 | 0.0 | 0.0 |
| *Myotis tricolor* (♂) | 22.7 | 0.0 | 0.0 | 0.0 | 5.3 | 0.0 | 0.5 | 0.0 | 0.0 | 0.0 |
| Benthic algae | 10.7 | 4.8 | 12.9 | 2.4 | 1.1 | 0.1 | 1.1 | 1.0 | 0.3 | 0.2 |
| Terrestrial | 14.9 | 5.3 | 22.8 | 12.2 | 0.2 | 0.1 | 0.0 | 0.0 | 0.0 | 0.0 |
|  |  |  |  |  |  |  |  |  |  |  |
| **Kalkoenkrans** |  |  |  |  |  |  |  |  |  |  |
| *Miniopterus natalensis* (♀) | 5.6 | 3.1 | 5.6 | 2.5 | 1.6 | 1.0 | 0.6 | 0.4 | 0.0 | 0.0 |
| *Miniopterus natalensis* (♂) | 5.7 | 3.5 | 1.8 | 2.0 | 1.5 | 0.8 | 0.3 | 0.1 | 0.0 | 0.0 |
| *Myotis tricolor* (♀) | 9.0 | 5.5 | 1.1 | 1.9 | 0.8 | 1.0 | 0.3 | 0.1 | 0.6 | 0.4 |
| *Myotis tricolor* (♂) | 3.1 | 0.0 | 0.0 | 0.0 | 0.3 | 0.0 | 0.5 | 0.0 | 0.6 | 0.0 |
| Benthic algae | 10.7 | 4.8 | 12.9 | 2.4 | 1.1 | 0.1 | 1.1 | 1.0 | 0.3 | 0.2 |
| Terrestrial | 14.9 | 5.3 | 22.8 | 12.2 | 0.0 | 0.1 | 0.0 | 0.0 | 0.0 | 0.0 |
|  |  |  |  |  |  |  |  |  |  |  |
| **Bazley** |  |  |  |  |  |  |  |  |  |  |
| *Miniopterus natalensis* (♀) | 5.3 | 1.9 | 0.6 | 0.7 | 0.6 | 0.3 | 0.2 | 0.1 | 0.3 | 0.2 |
| *Miniopterus natalensis* (♂) | 7.5 | 1.1 | 0.3 | 0.4 | 0.9 | 0.5 | 0.1 | 0.1 | 0.2 | 0.1 |
| *Myotis tricolor* (♀) | 9.4 | 0.0 | 0.0 | 0.0 | 0.9 | 0.0 | 0.0 | 0.0 | 0.0 | 0.0 |
| Benthic algae | 7.1 | 1.0 | 30.0 | 19.6 | 0.7 | 0.5 | 1.1 | 0.7 | 1.2 | 1.3 |
| Terrestrial | 20.9 | 2.0 | 45.1 | 3.3 | 0.1 | 0.1 | 0.0 | 0.0 | 0.0 | 0.0 |
