# Supplementary material for: Faecal analyses and alimentary tracers reveal the foraging ecology of two sympatric bats
Source: PLoS One. 2020 Jan 16;15(1):e0227743. doi: 10.1371/journal.pone.0227743 (PMC6964858; doi:10.1371/journal.pone.0227743)
Supplement: S6 Table — Mean percentage contributions (credibility intervals displayed in parentheses) of food sources from Bayesian mixing models (MixSIAR). Stable isotope mixing models were based on insect prey while fatty acid mixing models were based on benthic algae and terrestrial plants as proxies for aquatic and terrestrial insects. See S2 Table for the arthropod taxa placed in the three categories: aquatic, terrestrial and trawled. (DOCX) [file pone.0227743.s006.docx]

**S6 Table. Results of Bayesian mixing models.** Mean percentage contributions (credibility intervals displayed in parentheses) of food sources from Bayesian mixing models (MixSIAR). Stable isotope mixing models were based on insect prey while fatty acid mixing models were based on benthic algae and terrestrial plants as proxies for aquatic and terrestrial insects. See S2 Table for the arthropod taxa placed in the three categories: aquatic, terrestrial and trawled.

| Site | Stable isotope mixing models | | |  | Fatty acids mixing models | |
| --- | --- | --- | --- | --- | --- | --- |
|  | **Aquatic** | **Terrestrial** | **Trawled** |  | **Aquatic** | **Terrestrial** |
| **De Hoop** |  |  |  |  |  |  |
| *Miniopterus natalensis (♀)* | 2 (0-22) | 96(69-100) | 1 (0-15) |  | 67 (71-84) | 33 (16-40) |
| *Miniopterus natalensis (♂)* | 39 (0-100) | 54.6(0-100) | 7(0-16) |  | 68(74-90) | 32 (14-36) |
| *Myotis tricolor (♀)* | 23 (0-100) | 42(0-100) | 35(35-100) |  | 93(77-99) | 7(1-23) |
| *Myotis tricolor (♂)* | 21(0-100) | 31(0-100) | 48(39-100) |  | 92(77-99) | 8(2-23) |
|  |  |  |  |  |  |  |
| **Algeria** |  |  |  |  |  |  |
| *Miniopterus natalensis (♀)* |  |  |  |  |  |  |
| *Miniopterus natalensis (♂)* | 20 (0-100) | 74(0-100) | 6(0-42) |  | 45(2-96) | 55 (4-98) |
| *Myotis tricolor (♀)* | 46(0-100) | 26(0-100) | 28(0-100) |  | 95(80-100) | 5(2-20) |
| *Myotis tricolor (♂)* | **No data** | | |  | **No data** | |
|  |  |  |  |  |  |  |
| **Kalkoenkrans** |  |  |  |  |  |  |
| *Miniopterus natalensis (♀)* | 19 (0-100) | 69(12-100) | 12(0-100) |  | 26(23-29) | 74(71-77) |
| *Miniopterus natalensis (♂)* | 4(0-43) | 93(30-100) | 3(0-28) |  | 15(12-18) | 85(82-88) |
| *Myotis tricolor (♀)* | 29(0-100) | 39(0-100) | 31(5-100) |  | 91 (52-99) | 9 (0-48) |
| *Myotis tricolor (♂)* | 40(0-100) | 17(0-100) | 43(40-100) |  | 91 (53-100) | 9 (1-48) |
|  |  |  |  |  |  |  |
| **Sudwala** |  |  |  |  |  |  |
| *Miniopterus natalensis (♀)* | 1(0-11) | 98(85-100) | 1(0-8) |  | 3(3-8) | 97(92-100) |
| *Miniopterus natalensis (♂)* | 1(0-11) | 98(84-100) | 1(0-9) |  | 5(5-10) | 95(87-100) |
| *Myotis tricolor (♀)* | 1(0-8) | 99(90-100) | 0(0-5) |  | 2(1-7) | 98(99-100) |
| *Myotis tricolor (♂)* | **No data** | | |  | **No data** | |
|  |  |  |  |  |  |  |
| **Bazley** |  |  |  |  |  |  |
| *Miniopterus natalensis (♀)* | 3(0-26) | 94(37-100) | 3(0-35) |  | 32(0-98) | 68(87-100) |
| *Miniopterus natalensis (♂)* | 4(0-37) | 93(22-100) | 4(0-38) |  | 34(16-100) | 66(84-100) |
| *Myotis tricolor (♀)* | 4(0-52) | 93(5-100) | 4(0-50) |  | 36(16-99) | 64(84-100) |
| *Myotis tricolor (♂)* | **No data** | | |  | **No data** | |
